# Identification of potential microRNAs and KEGG pathways in denervation muscle atrophy based on meta-analysis

**DOI:** 10.1038/s41598-021-92489-1

**Published:** 2021-06-30

**Authors:** Xinyi Gu, Bo Jin, Zhidan Qi, Xiaofeng Yin

**Affiliations:** 1 Department of Orthopedics and Traumatology, Peking University People’s Hospital, Beijing, 100044 China; 2 Key Laboratory of Trauma and Neural Regeneration (Peking University), Beijing, 100044 China

**Keywords:** Cell biology, Genetics

## Abstract

The molecular mechanism of muscle atrophy has been studied a lot, but there is no comprehensive analysis focusing on the denervated muscle atrophy. The gene network that controls the development of denervated muscle atrophy needs further elucidation. We examined differentially expressed genes (DEGs) from five denervated muscle atrophy microarray datasets and predicted microRNAs that target these DEGs. We also included the differentially expressed microRNAs datasets of denervated muscle atrophy in previous studies as background information to identify potential key microRNAs. Finally, we compared denervated muscle atrophy with disuse muscle atrophy caused by other reasons, and obtained the Den-genes which only differentially expressed in denervated muscle atrophy. In this meta-analysis, we obtained 429 up-regulated genes, 525 down-regulated genes and a batch of key microRNAs in denervated muscle atrophy. We found eight important microRNA-mRNA interactions (miR-1/Jun, miR-1/Vegfa, miR-497/Vegfa, miR-23a/Vegfa, miR-206/Vegfa, miR-497/Suclg1, miR-27a/Suclg1, miR-27a/Mapk14). The top five KEGG pathways enriched by Den-genes are Insulin signaling pathway, T cell receptor signaling pathway, MAPK signaling pathway, Toll-like receptor signaling pathway and B cell receptor signaling pathway. Our research has delineated the RNA regulatory network of denervated muscle atrophy, and uncovered the specific genes and terms in denervated muscle atrophy.

## Introduction

Denervated muscle atrophy refers to muscle atrophy caused by injury of nerves that innervate the muscle. Denervated muscle atrophy is the main cause of motor function loss after peripheral nerve injury. Due to the slow speed of nerve regeneration, it is difficult for regenerated axons to reach the target muscle in a short time. Skeletal muscle will atrophy and fibrosis, and eventually lead to poor recovery of muscle function or even failure to recover^1–3^. Over time, due to muscle fiber necrosis, connective tissue hyperplasia, muscle cell regeneration failure and a large loss of muscle cell, the muscles at nerve endings lose the ability to accept the regenerated motor axons^4^. In addition, depletion of skeletal muscle satellite cells, changes in related protein metabolism and enzyme activities, vascular bed remodeling and regulation of myogenic factors are all important reasons for denervated muscle atrophy^5–9^.

At present, the main methods of treating denervated muscle atrophy include electrical stimulation^10,11^, passive exercise^12^, and various drug treatments. Up-regulated breakdown of protein in skeletal muscle is a sign of atrophy, so all potential drugs target the proteolytic system to cure or prevent atrophy^13^. Nevertheless, the FDA has not recommended other drugs to treat muscle atrophy except for megestrol acetate (MA)^14^, because these drugs cannot effectively target whole proteolytic system, and the potential targets of denervated muscle atrophy have not been fully explored.

The balance between protein synthesis and degradation maintains muscle mass^15,16^. In atrophic muscles, muscle-specific E3 ubiquitin ligase and muscle-specific ring finger 1 (MuRF1) in the ubiquitin proteasome system (UPS) are activated, promoting protein degradation in an adenosine triphosphate-dependent manner^17,18^. The biological axis composed of histone deacetylases (HDACs) 4 and 5, transcription factors Dach2 and myogenin can control the expression of many denervation related genes, including atrogin and MuRF1^19–22^. Myogenin directly mediates the transcriptional activation of the gene encoding E3 ubiquitin ligase, and the lockdown of HDAC4, HDAC5 or myogenin can alleviate denervated muscle atrophy^23^. In addition, the FoxO family is also an important regulator of atrogin and MuRF1. Overexpression of FoxOs can induce gene expression of atrogin and MuRF1, which will eventually lead to muscle atrophy^24,25^. Considering the complexity of these processes leading to denervated muscle atrophy, the identification of genes and pathways based on meta-analysis may help to understand the underlying molecular mechanisms.

Denervation, long-term bed rest, unloading of hind limbs, immobilization or microgravity can cause disuse muscle atrophy, of which the common features are decreased cross-sectional area of muscle fibers, decreased muscle strength, increased insulin resistance, and the transition of fiber types from slow to fast^26^. Decreased protein synthesis and increased protein degradation are the main reasons for the rapid loss of muscle protein due to denervation^26^. Current research focuses on the general signaling pathways and common key regulators that mediate disuse muscle atrophy^26–28^, but obviously, denervation is also accompanied by the loss of neurotrophic factors and a series of changes brought about by nerve injury. These denervation-specific pathological changes might be accompanied by specific transcriptome changes which remain to be discovered.

We systematically searched and integrated the gene expression data of denervated muscle atrophy to construct the microRNA-mRNA regulatory network. By comparing denervated muscle atrophy with disused muscle atrophy caused by other reasons, we have discovered the genes and terms that are specifically differentially expressed in denervated muscle atrophy.

## Results

### Data screening and inclusion criteria

**Figure 1:**
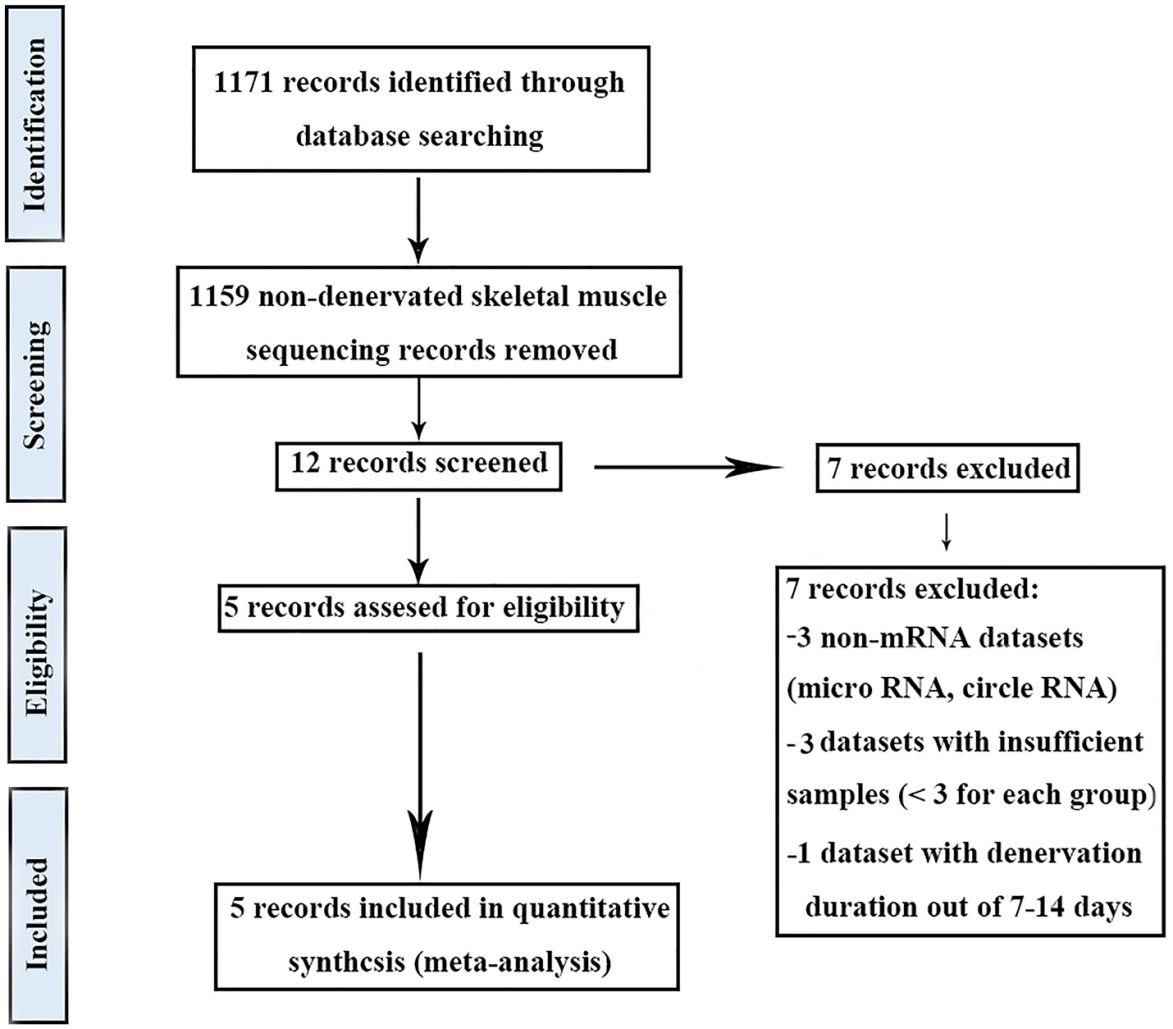
Flow chart of literature search in the meta-analysis.

This study obtained five gene expression data of denervated muscle atrophy after screening (Fig. 1)^29–31^. The details of these data were aggregated into a table (Table 1) and they were all from mouse models in different muscles, including tibialis anterior muscle, gastrocnemius muscle, and gastrocnemius triceps muscle. We only included the data sets that denervated for 7–14 days, because the muscle with denervation greater than 7 days has entered the stage of pathological atrophy^31^, and the gene expression profiles denervated for 7/14 days have high similarity, which can ensure the homogeneity of the included data sets^32^.

**Table 1:** Description of publicly available data sets used in the meta-analysis.

Number	Gene expression platforms	Muscle	Species	Intervention duration	Ref.
1	Illumina mouse-6 v1.1	Triceps surae muscle	Mus Musculus	14 days	GSE44205^29^
2	Affymetrix Mouse Exon 1.0 ST Array	Tibialis anterior muscles	Mus Musculus	7 days	GSE39195
3	Agilent-014868	Tibialis anterior muscles	Mus Musculus	14 days	GSE49826^30^
4	Illumina HiSeq X	Gastrocnemius muscle	Mus Musculus	7 days	^31^
5	Illumina HiSeq X	Gastrocnemius muscle	Mus Musculus	14 days	^31^

*Ref* reference.

### DEGs in denervated muscle atrophy

**Figure 2:**
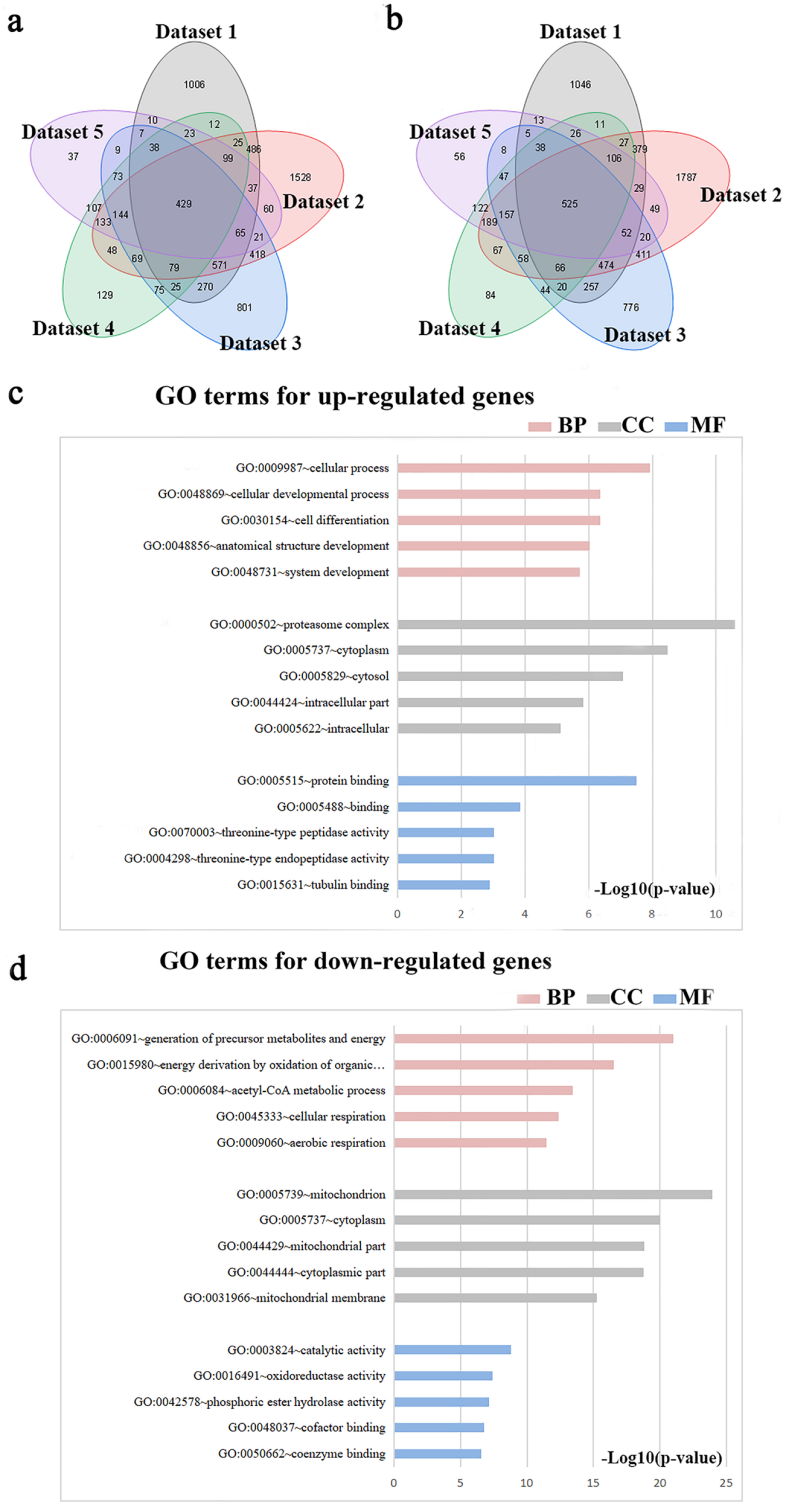
DEGs and GO terms in denervated muscle atrophy were identified. Venn diagrams for (**a**) up-regulated mRNAs and (**b**) down-regulated mRNAs were drawn based on 5 denervated muscle atrophy datasets. GO analysis of DEGs in denervated muscle atrophy were conducted for (**c**) up- and (**d**) down-regulated mRNAs respectively (Each figure shows the top10 terms with the lowest p value). *BP* biological process, *CC* cellular component, *MF* molecular function.

By crossing five data sets, we obtained 429 up-regulated mRNAs and 525 down-regulated mRNAs (Fig. 2a,b) (Table S1), GO analysis revealed the biological role of DEGs in denervated muscle atrophy (Table S2). The analysis showed the pathways enriched by up-regulated genes included cellular process, cellular developmental process, cell differentiation, anatomical structure development, system development, proteasome complex, cytoplasm, cytosol, intracellular part, intracellular, protein binding, binding, threonine-type peptidase activity, threonine-type endopeptidase activity and tubulin binding. The pathways enriched by down-regulated genes include generation of precursor metabolites and energy, energy derivation by oxidation of organic compounds, acetyl-CoA metabolic process, cellular respiration, aerobic respiration, mitochondrion cytoplasm, mitochondrial part, cytoplasmic part, mitochondrial membrane, catalytic activity, oxidoreductase activity, phosphoric ester hydrolase activity, cofactor binding and coenzyme binding (Fig. 2c,d). In order to avoid losing information, we performed GO analysis on the DEG of each data set, and then obtained GO terms that were commonly enriched in each data set (Table 2). We found that the up-regulated GO terms were mostly concentrated in cellular components, and the down-regulated GO terms were still dominated by energy metabolism.

**Table 2:** GO terms commonly altered in five data sets.

	Up-regulated GO terms	Down-regulated GO terms
BP	GO:0071840 ~ cellular component organization or biogenesis	GO:0044710 ~ single-organism metabolic process
	GO:0016043 ~ cellular component organization	GO:0055114 ~ oxidation–reduction process
	GO:0051128 ~ regulation of cellular component organization	GO:0044281 ~ small molecule metabolic process
	GO:0044267 ~ cellular protein metabolic process	GO:0015980 ~ energy derivation by oxidation of organic compounds
	GO:0048522 ~ positive regulation of cellular process	GO:0006091 ~ generation of precursor metabolites and energy
	GO:0044424 ~ intracellular part	GO:0005737 ~ cytoplasm
	GO:0005622 ~ intracellular	GO:0043227 ~ membrane-bounded organelle
CC	GO:0043229 ~ intracellular organelle	GO:0044444 ~ cytoplasmic part
	GO:0043226 ~ organelle	GO:0043226 ~ organelle
	GO:0043227 ~ membrane-bounded organelle	GO:0044424 ~ intracellular part
	GO:0005515 ~ protein binding	GO:0005515 ~ protein binding
	GO:0005488 ~ binding	GO:0003824 ~ catalytic activity
MF	GO:0019899 ~ enzyme binding	GO:0019899 ~ enzyme binding
	GO:0044822 ~ poly(A) RNA binding	GO:0005488 ~ binding
	GO:0044877 ~ macromolecular complex binding	GO:0048037 ~ cofactor binding

*BP* biological process, *CC* cellular component, *MF* molecular function.

**Figure 3:**
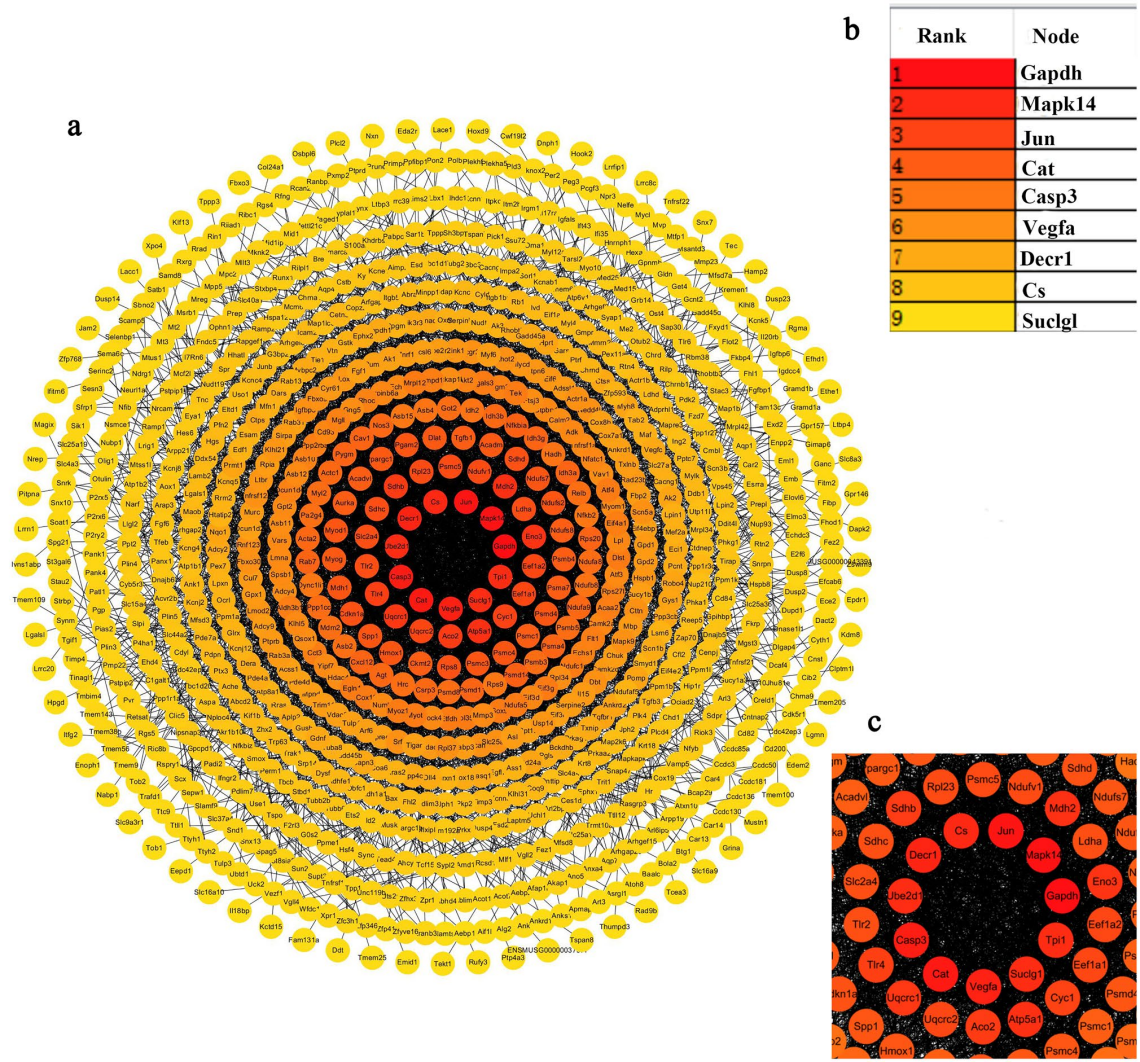
PPI network in denervated muscle atrophy. (**a**) PPI network, the color was used to reflect the level of degree, the higher the degree, the darker the node color. (**b**) The top10 DEGs with the highest degree. (**c**) Partial enlarged view of PPI network. STRING v11.0 was used to generate protein interactions, and the resulting network was visualized using Cytoscape v3.7.2.

A protein–protein Interaction (PPI) network was mapped based on DEGs to bring in more function-related proteins, and the nodes were sorted according to the degree of interaction between the nodes (Fig. 3). The top10 proteins are Gapdh, Mapk14, Jun, Cat, Casp3, Vegfa, Decr1, CS and Suclg1. The function and location of these genes were annotated respectively, and most of these genes are related to energy metabolism (Table 3).

**Table 3:** Description of top10 proteins in PPI.

Official symbol	Molecular function	Biological process	Location
Gapdh	Oxidoreductase, transferase	Apoptosis, glycolysis, translation regulation	Plasma membrane and cytosol
Mapk14	Kinase, serine/threonine-protein kinase, transferase	Apoptosis, stress response, transcription	Nuclear speckles and cytosol
Jun	Activator, DNA-binding	Transcription, transcription regulation	Nucleoplasm
Cat	Mitogen, oxidoreductase, peroxidase	Hydrogen peroxide	Vesicles
Casp3	Hydrolase, protease, thiol protease	Apoptosis	Nucleoplasm and mitochondria
Vegfa	Developmental protein, growth factor, Heparin-binding, Mitogen	Angiogenesis, differentiation	Secreted to blood
Decr1	Oxidoreductase	Fatty acid metabolism, lipid metabolism	Mitochondria and cytosol
Cs	Transferase	Tricarboxylic acid cycle	Mitochondria
Suclg1	Ligase	Tricarboxylic acid cycle	Mitochondria and plasma membrane
Tpi1	Isomerase, lyase	Gluconeogenesis, glycolysis	Nucleoplasm and vesicles

### Identification of potential microRNAs in denervated muscle atrophy

**Figure 4:**
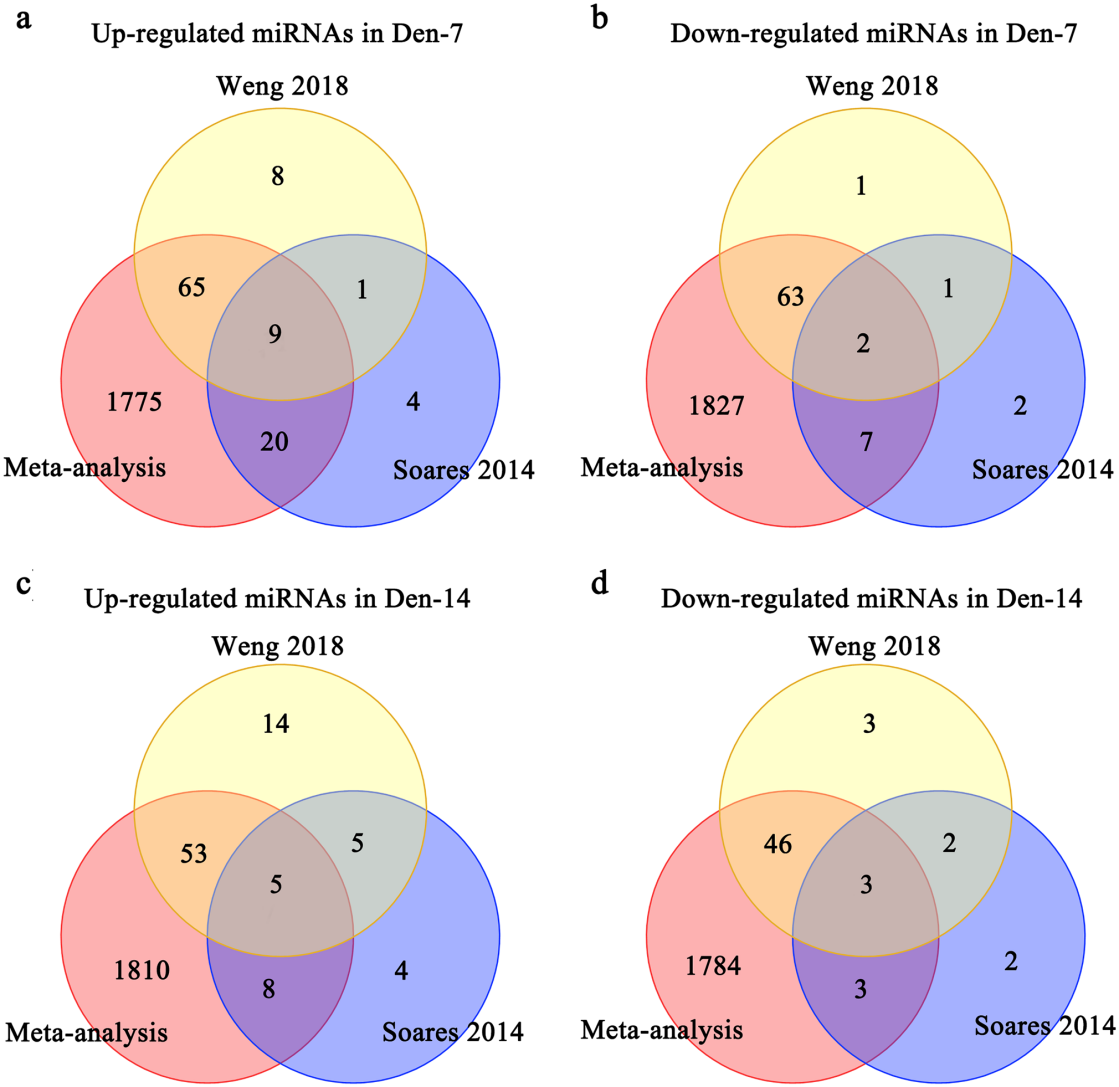
MicroRNAs identified in denervated muscle atrophy. MicroRNAs predicted as targeting mRNAs retrieved by our meta-analysis were further compared with microRNAs identified as differentially expressed in previous studies of denervated muscle atrophy for 7 days and 14 days respectively. (**a**) Venn for up-regulated miRNAs for Den-7 days. (**b**) Venn for down-regulated miRNAs for Den-7 days. (**c**) Venn for up-regulated miRNAs for Den-14 days. (**d**) Venn for down-regulated miRNAs for Den-14 days. *Den-7 days* denervation for 7 days, *Den-14 days* denervation for 14 days.

MiRNA-mRNA target prediction identified miRNAs targeting down-regulated mRNAs and those targeting up-regulated mRNAs for den-7 days and den-14 days respectively. By retrieving, we obtained microRNA data sets denervated 7/14 days^31,32^, we intersected the up-regulated miRNAs in these two data sets with the miRNAs targeting down-regulated mRNAs in this Meta-analysis to obtain 9 miRNAs for den-7 days. Similarly, we obtained 2 down-regulated miRNAs for den-7 days, 5 up-regulated miRNAs and 3 down-regulated miRNAs for den-14 days (Fig. 4, Table 4). We predicted the target genes of these key miRNAs and found eight microRNA-mRNA interactions of which the mRNAs are the top 10 denervation-related mRNAs identified in this research (miR-1/Jun, miR-1/Vegfa, miR-497/Vegfa, miR-23a/Vegfa, miR-206/Vegfa, miR-497/Suclg1, miR-27a/Suclg1, miR-27a/Mapk14). Notably, seven interactions showed an opposite direction of expression between microRNAs and mRNAs (Table 5).

**Table 4:** List of key miRNAs in denervated muscle atrophy.

Den 7	Den 14
mmu-miR-23a↑	mmu-miR-27b↑
mmu-miR-497↑	mmu-miR-27a↑
mmu-miR-199b↑	mmu-miR-24↑
mmu-miR-199a↑	mmu-miR-21↑
mmu-miR-24↑	mmu-miR-206↑
mmu-miR-27b↑	
mmu-miR-27a↑	
mmu-miR-206↑	
mmu-miR-21↑	
mmu-miR-30c↓	mmu-miR-30c↓
mmu-miR-30b↓	mmu-miR-497↓
	mmu-miR-1↓

↓, down regulated; ↑, up regulated.

*Den 7* denervation for 7 days; *Den 14* denervation for 14 days.

**Table 5:** MicroRNA/mRNA interactions.

MicroRNA	mRNA
mmu-miR-1↓	Jun↑
mmu-miR-1↓	Vegfa↓
mmu-miR-497↑	Vegfa↓
mmu-miR-23a↑	Vegfa↓
mmu-miR-206↑	Vegfa↓
mmu-miR-497	Suclg1↓
mmu-miR-27a↑	Suclg1↓
mmu-miR-27a↑	Mapk14↓

↓, down regulated; ↑, up regulated.

### Specific genes in denervated muscle atrophy

**Figure 5:**
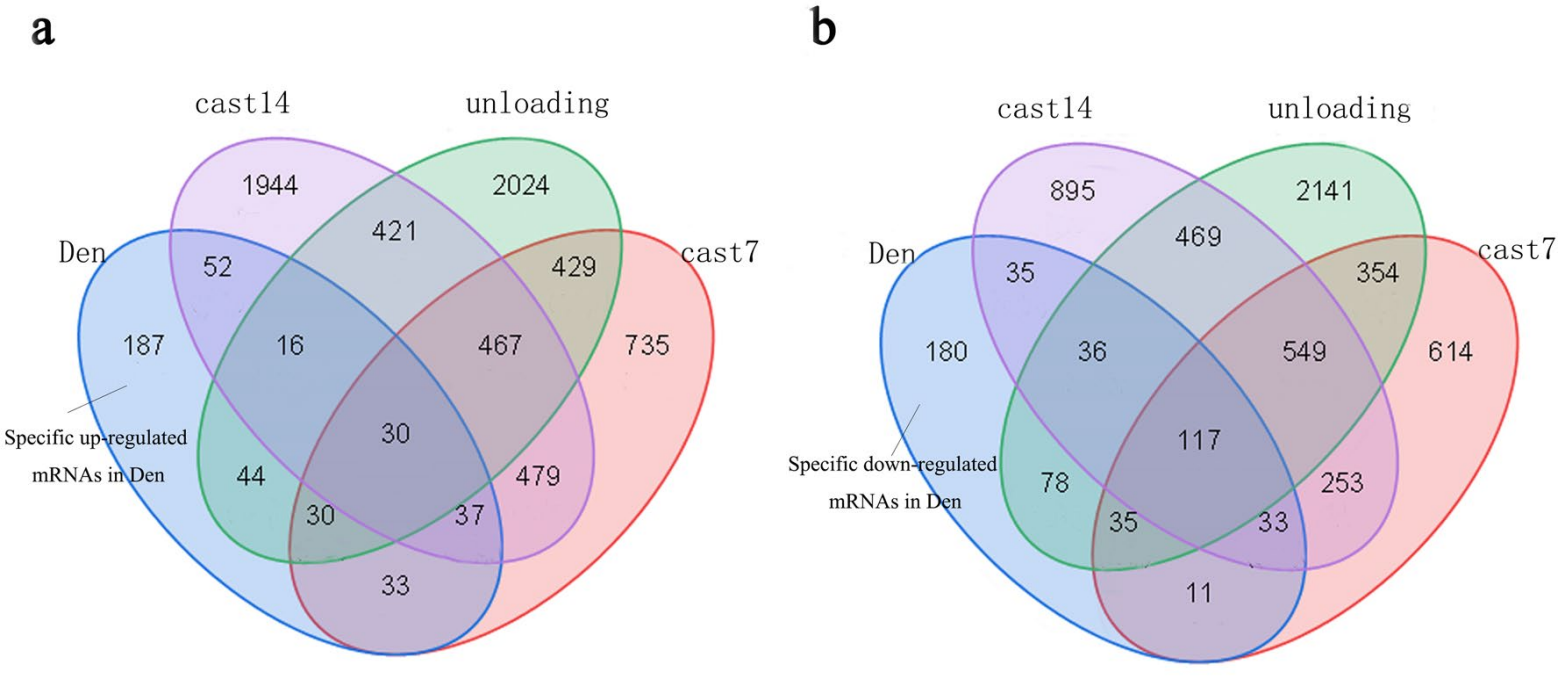
Specific mRNAs identified in denervated muscle atrophy. DEGs in denervated muscle atrophy retrieved by our meta-analysis (blue circle) were further compared with DEGs in disuse muscle atrophy caused by 7-day hindlimb casting (purple circle), 14-day hindlimb casting (red circle) and hindlimb unloading (green circle). (**a**) Venn for up-regulated genes. (**b**) Venn for down-regulated genes. *Den* denervation, *cast 7* 7-day hindlimb casting, *cast 14* 14-day hindlimb casting.

**Figure 6:**
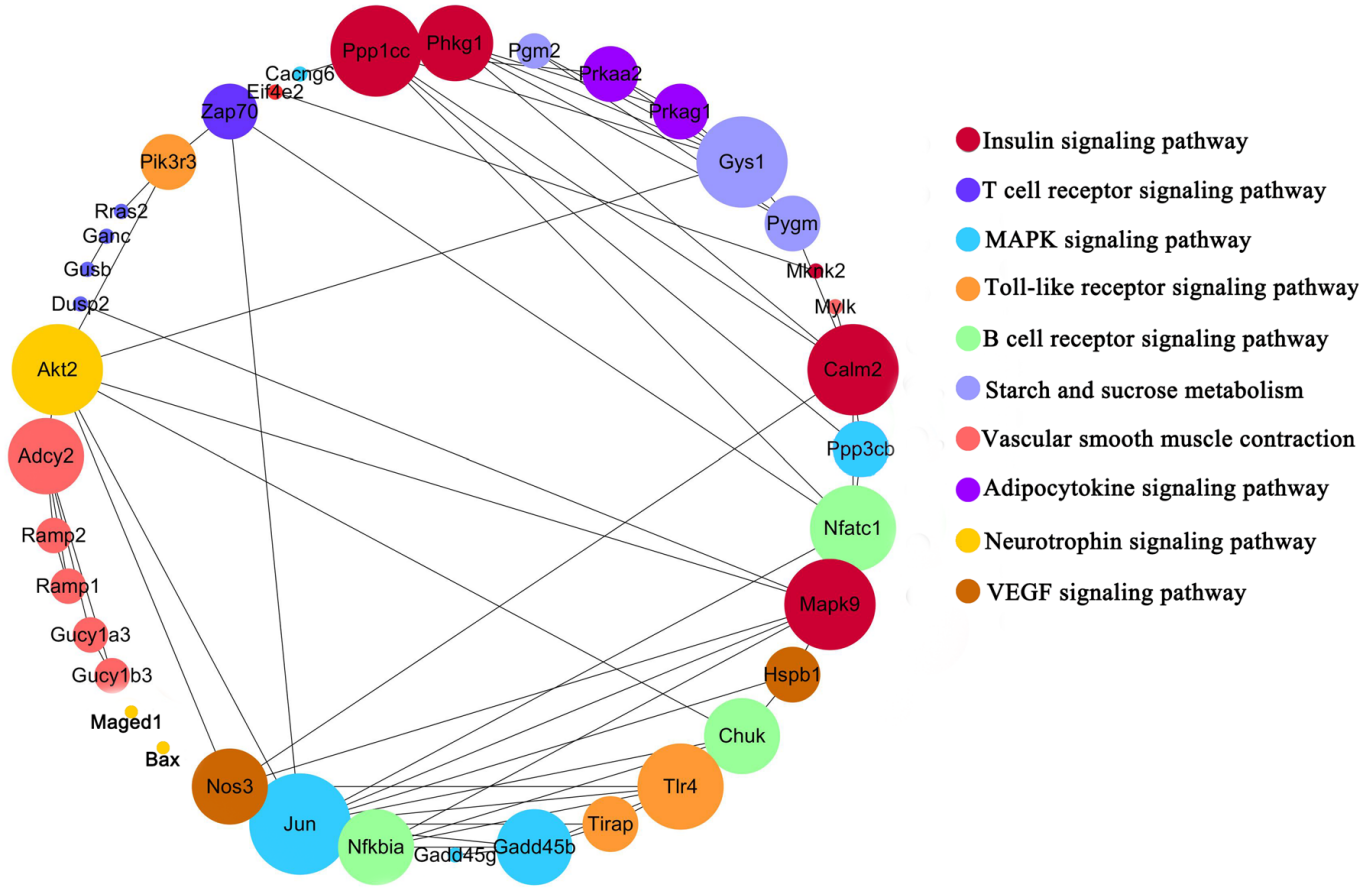
KEGG analysis of den-genes. Lines highlight KEGG network, with Jun, Mapk9, Calm2, Gys1, Ppp1cc, and Akt2 presenting the highest degree of interactions. Colors highlight KEGG pathways of the circle network components. The larger the circle, the higher the interaction degree identified. Cytoscape v. 3.7.2 was used to generate the resulting network. Permission has been obtained from Kanehisa laboratories for using KEGG pathway database^33^.

By retrieving, we got three data sets of disuse muscle atrophy caused by unloading or casting (Table 6), genes only differentially expressed in denervated muscle atrophy were regarded as denervation specific genes, called den-genes. We got 187 up-regulated den-genes and 180 down-regulated den-genes (Fig. 5), then performed KEGG enrichment analysis on these genes and got top10 most enriched pathways (Fig. 6). We searched and found the expression of neurotrophic factors (NGF, BDNF, NT-3, NT-4, CNTF, Neuregulin-1 and Neuritin) changed in nerves or muscles after nerve injury and there was evidence that Insulin signaling pathway, MAPK signaling pathway, Neurotrophin signaling pathway, T cell receptor signaling pathway, Toll-like receptor signaling pathway, and B cell receptor signaling pathway were regulated by the above neurotrophic factors (Table 7). Furthermore, most of these terms in skeletal muscle showed different expression after electrical stimulation and Insulin signaling pathway, MAPK signaling pathway, Toll-like receptor signaling pathway, Neurotrophin signaling pathway, and VEGF signaling pathway show regulatory effects on ion channels and connexins (Table 8).

**Table 6:** Description of publicly available datasets of disuse muscle atrophy.

Authors	Platforms	Muscle	Organism	Experiment	Intervention duration	Ref.
Zhang et al. 2018	Illumina HiSeq 2500	Soleus muscle	Mus Musculus	Unloading	10 days	GSE102284
Jelinsky et al. 2011	Affymetrix Mouse Genome 430	Gastrocnemius	Mus Musculus	Casting	7 days	GSE25908
Jelinsky et al. 2011	Affymetrix Mouse Genome 430	Gastrocnemius	Mus Musculus	Casting	14 days	GSE25908

*Ref* reference.

**Table 7:** The function of key microRNAs in skeletal muscle.

MicroRNA	Function
mmu-miR-23a	Muscle atrophy (−)^34^
mmu-miR-497	Myoblast proliferation (−)^35^, myogenic differentiation (+)^35^
mmu-miR-199a	Slow-to-fast muscle fiber type conversion (+)^36^
mmu-miR-24	Myoblast proliferation (−)^37^, myogenic differentiation (+)^37^
mmu-miR-27b	Skeletal muscle satellite cells proliferation (−) and differentiation (+)^38^
mmu-miR-27a	Skeletal muscle lipid use (+)^39^, muscle atrophy (+)^40^
mmu-miR-206	Myogenic differentiation (+)^41^, neuromuscular synapses regeneration (+)^42^
mmu-miR-21	Myogenic differentiation (+)^43^
mmu-miR-497	Skeletal muscle stem cells proliferation (−)^44^, myogenic differentiation (+)^35^
mmu-miR-1	Myogenesis (+)^45^

+ positive regulation, − negative regulation.

**Table 8:** Altered neurotrophic factor expression in injured neurons or muscle.

Neurotrophic factor	Source	Expression	Downstream pathway
NGF	Neurons^46^, satellite glial cell^47^, Schwann cell^48^, Skeletal muscle^49^	Up	Insulin signaling pathway (IE), MAPK signaling pathway (DE)^50^, Neurotrophin signaling pathway (DE)
BDNF	Skeletal muscle^51^, Schwann cell^52^	Up	MAPK signaling pathway (DE)^53^, Neurotrophin signaling pathway (DE)
NT-3, NT-4	Neurons^54^, Skeletal muscle^55^	Up	MAPK signaling pathway (DE)^56^, Neurotrophin signaling pathway (DE)
CNTF	Schwann cell^57^	Down	T cell receptor signaling pathway (IE), Toll-like receptor signaling pathway (IE), B cell receptor signaling pathway (IE)^58^
Neuregulin-1	Schwann cell^59^	Up	MAPK signaling pathway (DE)^60^
Neuritin	Neurons^61^	Up	Insulin signaling pathway (IE)^62^, MAPK signaling pathway (IE)^62^

*NGF* nerve growth factor, *BDNF* brain-derived neurotrophic factor, *NT* neurotrophin, *DE* direct evidence, *IE* indirect evidence.

## Discussion

In this study, we collected and aggregated gene expression data sets of denervated muscle atrophy, and obtained 429 up-regulated mRNAs and 525 down-regulated mRNAs. We drew a PPI network, and sorted the nodes according to the interaction degree. The top10 proteins were Gapdh, Mapk14, Jun, Cat, Casp3, Vegfa, Decr1, Cs and Suclg1. Mapk14, Jun, and Casp3 have been confirmed to be important in denervated muscle atrophy. (1) Mapk14: the three main categories of mitogen-activated protein kinase (MAPK) family proteins are as follows: extracellular signal-regulated kinase (ERK), c-Jun N-terminal kinase (JNK) and p38 MAPK (Mapk14). The members of the p38 MAPK family (p38α, p38β, p38γ and p38δ MAPK) act as transducers of cellular stress and various non-stress-related stimuli. Therefore, the p38 MAPK pathway has multiple functions and is involved in various cellular processes, including aging, apoptosis, cell cycle arrest, inflammation, and tumorigenesis^63^. The p38 MAPK pathway can mediate the expression of MuRF1 and Atrogin1^64,65^, and knockdown of p38αMAPK can inhibit muscle atrophy caused by denervation^66^. (2) Jun: the protein deacetylase HDAC4 is strongly induced in muscles affected by motor neuron diseases such as ALS^19^. Denervation-induced HDAC4 activates AP1 (Jun) transcription factor by stimulating MAPK signaling to promote denervated muscle atrophy^67^. (3) Casp3: in the ubiquitin–proteasome pathway and apoptosis process, the activation of Caspase-3 (Casp3) is a common phenomenon. Caspase-3 regulates denervation-induced signal transduction through the mitochondrial-related cell death/apoptosis pathway, resulting in the loss of muscle mass. The lack of caspase-3 has a protective effect on denervated muscle atrophy^68^. The other proteins Gapdh, Cat, Vegfa, Decr1, Cs, and Suclg1 are mostly important links in redox and energy metabolism pathways^69–73^, and they may be potential targets in denervated muscle atrophy for treatment.

We included the differentially expressed miRNA expression data of denervated muscle atrophy in previous studies as background information^31,32^, identified the potential key microRNAs and eight microRNA-mRNA interactions of which the mRNAs are the top 10 denervation-related mRNAs identified in this research (miR-1/Jun, miR-1/Vegfa, miR-497/Vegfa, miR-23a/Vegfa, miR-206/Vegfa, miR-497/Suclg1, miR-27a/Suclg1, miR-27a/Mapk14). Most of the Key miRNAs we obtained showed important regulatory effects on skeletal muscle (Table 7). After 7-day hind limbs unloading in mice, the serum levels of muscle-specific miRNAs such as miR-1, miR-23a, miR-206 increased significantly, and could induce severe muscle atrophy^74^. MiR-1 has been confirmed to play an important role in skeletal muscle development^75,76^. MiR-23a can target Atrogin-1 and MuRF1 and inhibit their translation, and the ectopic expression of miR-23a can protect muscles from atrophy in vitro and in vivo, indicating that miR-23a is a key regulator of muscle atrophy^77^. Overexpression of miR-27a in mice with chronic kidney disease attenuated muscle loss, improved grip strength and reduced the expression of FoxO1, MuRF1and Atrogin1^40^.

Denervation, long-term bed rest, unloading of hind limbs, immobilization or microgravity can cause disuse muscle atrophy. Current research focuses on the general signaling pathways and common key regulators that mediate disuse muscle atrophy^26–28^, but obviously, denervation is also accompanied by the loss of neurotrophic factors and a series of changes brought about by nerve injury. These denervation-specific pathological changes might be accompanied by specific transcriptome changes which remain to be discovered.

The nervous system controls skeletal muscle through two mechanisms: (1) neuromotor control, which causes muscle contraction through excitation and contraction coupling. (2) Neurotrophic control, which regulates muscles by releasing soluble factors from the nerve endings of motor neurons on the NMJ (Neuromuscular junction)^78^. NGF, BDNF, NT-3, NT-4, CNTF, Neuregulin-1 and Neuritin are neurotrophic factors differentially expressed in nerves or skeletal muscle after nerve injury^79^, and they were found to regulate Insulin signaling pathway, MAPK Signaling pathway, Neurotrophin signaling pathway, T cell receptor signaling pathway, Toll-like receptor signaling pathway and B cell receptor signaling pathway which enriched by den-genes (Table 8). The specific changes in skeletal muscle after nerve transection include increased membrane permeability, decreased membrane potential and increased membrane excitability. Most of these are caused by changes in the expression of ion channels and the insertion of connexins 39, 43, and 45 into the muscle membrane, which mediate skeletal muscle atrophy^78,80^. Insulin signaling pathway, MAPK signaling pathway, Toll-like receptor signaling pathway, Neurotrophin signaling pathway and VEGF signaling pathway showed the potential to regulate ion channels and connexins. Insulin signaling pathway and Toll-like receptor signaling pathway can affect the formation of neuromuscular junctions (Table 9). Therefore, Insulin signaling pathway and Toll-like receptor signaling pathway, as metabolic or inflammation-related terms, have been seldom studied in the neuromuscular systerm, but this study found that they may have important research value in the denervated muscle atrophy.

**Table 9:** The regulation of KEGG pathways enriched by den-genes on muscle components.

Pathway	Altered after ECS^81^	NMJ	Ion channel	Connexins
Insulin signaling pathway	+	+ ^82^	Kv4.2^83^	−
T cell receptor signaling pathway	+	−	−	−
MAPK signaling pathway	+	+ ^84^	K _ATP_^85^, Kv2.1^86^, Nav1.5^87^	Connexin 43^88^
Toll-like receptor signaling pathway	+	+ ^89^	Kv4.2/4.3^90^, TRPV1^91^, K _ATP_^85^	Connexin 43^92^
B cell receptor signaling pathway	+	−	−	−
Starch and sucrose metabolism	+	−	−	−
Vascular smooth muscle contraction	−	−	−	−
Adipocytokine signaling pathway	−	−	−	−
Neurotrophin signaling pathway	+	+ ^93^	CLC-4^94^, Kv1.3^95^	−
VEGF signaling pathway	+	−	TRPV4^96^, TRPM7	−

*NMJ* neuromuscular junction, *ECS* electrical Stimulation, *CLC-4* chloride channel 4, *TRPV4* transient receptor potential vanilloid 4, *TRPM7* transient receptor potential melastatin-subfamily 7.

Current research focuses on the general signaling pathways and common key regulators that mediate disuse muscle atrophy, but obviously, denervation is also accompanied by the loss of neurotrophic factors and a series of changes brought about by nerve injury. These denervation-specific pathological changes must be accompanied by specific transcriptome changes which remain to be discovered. We systematically searched and integrated the gene expression data of denervated muscle atrophy to construct the microRNA-mRNA regulatory network. By comparing denervated muscle atrophy with disused muscle atrophy caused by other reasons, we have discovered the genes and terms that are specifically differentially expressed in denervated muscle atrophy.

## Methods

### Inclusion criteria for gene expression data

We performed a meta-analysis following the PRISMA Statement^97^, and found the gene expression data of denervated muscle atrophy by searching NCBI-GEO (http://www.ncbi.nlm.nih.gov/geo/) and PubMed (http://www.ncbi.nlm.nih.gov/pubmed). The keywords used were: “Denervated atrophy”, “Denervation AND muscle”, “nerve AND muscle”, and “Denervation AND muscle AND sequencing”. These meta-analysis searches comprised studies published between 2010 and 2020. Our inclusion criteria were (1) gene expression data in skeletal muscle with peripheral nerve injury, (2) the number of samples in each group should be greater than two, (3) the duration of denervation was 7 to 14 days, (4) all types of skeletal muscle were considered, (5) the inclusion of normal tissues for comparison, and (6) all gene expression analysis platforms were considered. Our exclusion criteria were (1) non-muscle samples, (2) non-mRNA datasets, and (3) review studies.

And we also searched NCBI-GEO (http://www.ncbi.nlm.nih.gov/geo/) and PubMed (http://www.ncbi.nlm.nih.gov/pubmed) to find the gene expression data of disuse muscle atrophy. The keywords used were: “Disuse atrophy”, “Disuse AND muscle”, “unloading AND muscle”, and “cast AND muscle”. These meta-analysis searches comprised studies published between 2010 and 2020. Our inclusion criteria were (1) gene expression data in skeletal muscle with disuse atrophy, (2) the number of samples in each group should be greater than two, (3) the duration of intervention was 7 to 14 days, (4) all types of skeletal muscle were considered, (5) the inclusion of normal tissues for comparison, and (6) all gene expression analysis platforms were considered. Our exclusion criteria were (1) non-muscle samples, (2) non-mRNA datasets, and (3) review studies.

In this study, each individual dataset was processed using edgeR packages to compare control samples and experiment samples to screen DEGs. The Benjamini & Hochberg false discovery rate method was used for p-value adjustment, and the default adj-p-value significance level cut-off is 0.05.

### Meta-analysis of global gene expression data in denervated muscle atrophy

Meta-analysis was performed on the five datasets of denervated muscle atrophy using Vote counting generic ways of combining information^98^, and the DEGs that were common between all datasets were identified. The results were visualized using Venn diagrams by a graphing software (ORIGIN2019; OriginLab, Northampton, MA).

### GO and KEGG enrichment analysis of DEGs

The DEGs were used to identify over-represented gene ontology categories and KEGG pathways by using DAVID 6.7 (https://david-d.ncifcrf.gov/). The p value has been corrected using FDR (false discovery rate). GO/KEGG enrichment with p < 0.05 was regarded as statistically significant and those with number of genes < 10 were removed. Bar chart of GO terms was drawn by Microsoft Excel 2016. KEGG network was visualized by Cytoscape software v. 3.7.2 54.

### Protein–protein interaction (PPI) network construction for common DEGs

The DEGs were subjected to STRING v.11.0 database^99^ analysis to construct PPI networks. Minimum required interaction score was 0.4 (medium confidence). PPI networks were visualized by Cytoscape software v. 3.7.2^100^ and the degree which indicates the number of interactions of each node was calculated by cytoHubba^101^.

### Identification of microRNAs as potential modulators of DEGs

The DEGs identified in our meta-analysis were used for microRNA prediction by DIANA-Tarbase^102^ to identify potential regulators in denervated muscle atrophy. Visualization of microRNA-gene interaction networks were generated using Cytoscape v3.7.2^100^ and the degree which indicates the number of interactions of each node was calculated by cytoHubba^101^.

## Supplementary Information

Supplementary Table S1.

Supplementary Table S2.
